# Synovitis, Acne, Pustulosis, Hyperostosis, Osteitis (SAPHO): An Interesting Clinical Syndrome

**DOI:** 10.7759/cureus.10184

**Published:** 2020-09-01

**Authors:** Ali Hussain, Mohsin Gondal, Nizar Abdallah, Hira Yousuf, Mubashar Iqbal

**Affiliations:** 1 Acute Medicine, Pinderfields General Hospital, Wakefield, GBR; 2 Cardiology, The Mid Yorkshire Hospitals NHS Trust, Wakefield, GBR; 3 Endocrinology, Diabetes and Metabolism, Pinderfields General Hospital, Wakefield, GBR; 4 Oncology, Pinderfields General Hospital, Wakefield, GBR; 5 Respiratory Medicine, Sheffield Teaching Hospitals NHS Foundation Trust, Sheffield, GBR

**Keywords:** sapho syndrome, cutibacterium acnes, dmards, bullhead sign

## Abstract

Synovitis, acne, pustulosis, hyperostosis, and osteitis (SAPHO) syndrome is a rare disorder that classically involves the musculoskeletal system, i.e. bone, joint and skin. The exact pathogenesis of this syndrome is unknown. However, autoimmunity, infections, immune malfunction and genetic factors are attributed to its pathophysiology. Bone and joint involvements are the hallmark of SAPHO syndrome and not necessarily require cutaneous involvement at the time of diagnosis. X-ray of the affected joints could show osteitis with sclerosis. Anterior chest wall involvement particularly ''bullhead appearance'' seen on bone scan is a striking feature of the syndrome. Erythrocyte sedimentation rate (ESR) is usually elevated amongst the majority of patients. Diagnosis of SAPHO is always challenging and often delayed because of a multitude of symptoms. The mainstay of treatment is control of pain and inflammation with both non-steroidal anti-inflammatory drugs (NSAIDs) and rescue courses of systemic steroids. If failed to control symptoms with first-line agents and in those with severe disease, disease-modifying anti-inflammatory drugs (DMARDs) may be needed eventually. Despite a chronic inflammatory condition, it remains stable in the majority of cases. Here in this case report, we reiterate the importance of early recognition, timely diagnosis and prompt treatment initiation.

## Introduction

It was in 1987 that a group of French authors coined the acronym SAPHO (synovitis, acne, pustulosis, hyperostosis, osteitis), a rare disorder of unknown aetiology [1]. The explicit data on its prevalence are not readily available mostly because of underdiagnosis; however, it is estimated to be around 1/10,000 in Caucasians [2]. As its exact pathogenesis is not known, a number of genetic and environmental factors (e.g., infectious diseases) along with immune dysregulation have been proposed to contribute to disease susceptibility and development. It predominantly involves females with the age distribution of 30-50 years [3]. SAPHO syndrome has a wide array of clinical presentations, which may lead to a delay in diagnosis for several years. Often symptoms can be attributed to other conditions that make SAPHO a challenging diagnosis [3-5].

Common conditions like infections and malignancies may masquerade as SAPHO. Diagnosis is usually made in the clinical context of serositis coupled with radiological findings of sclerosis and exclusion of infections and other rheumatological disorders. In terms of treatment approach, there are no definitive guidelines or protocols due to the lack of randomized trials and wide variations in symptom presentation.

## Case presentation

A 52-year-old married female presented with a past medical history of hysterectomy (secondary to postpartum bleeding). She also had chronic recurrent episodes of pain in the neck, shoulder and back over the last 12 years. She was seen in several hospitals after being diagnosed to have an osteoporotic fracture of the thoracic spine on CT scan. Bone mineral density (BMD) scan showed osteoporosis and was commenced on denosumab (60 mg subcutaneously) as a treatment of osteoporosis. She received two doses of denosumab; however, her complaints of neck and shoulder pain never improved.

On examination, the patient looked well. General physical examination was unremarkable except tender points in the upper chest, back and hip joints. The patient denied any history of dermatological involvement. Her bowel habits were normal. There was no history of steroids administration, and she was clinically euthyroid. Her family history was also unremarkable.

Her baseline investigations are shown in Table 1. Full blood count, bone profile, vitamin D, parathyroid hormone (PTH) and autoimmune profile were normal; however, there was evidence of raised erythrocyte sedimentation rate (ESR 47, normal range <20 mm/hr).

**Table 1:** Baseline investigations of the patient WBC, white blood cell; ESR, erythrocyte sedimentation rate; PTH, parathyroid hormone; TSH, thyroid-stimulating hormone; FT4, free thyroxine; RA, rheumatoid arthritis; ANA, antinuclear antibody; HCV, hepatitis C virus

Lab parameter	Patient results	Normal range
Full blood count (FBC)		
-Hb	11.4	11.0-14.5 g/dL
-WBC	4.3	2.4-9.5 x 10^9^/L
-Platelets	334	150-450 x 10^9^/L
Inflammatory markers		
-ESR	47	<20 mm/hr
Bone profile		
-Albumin	49	35-52 g/L
-Alkaline phosphatase	46	35-104 U/L
-Calcium (albumin adjusted)	2.45	2.15-2.55 mmol/L
-Phosphorus	1.18	0.81-1.45 mmol/L
Vitamin D (25-0H)	117	>75-250 nmol/L
PTH	6.3	1.6-6.9 pmol/L
Thyroid function tests		
-TSH	0.89	0.27-4.20 mIU/L
-FT4	20	13.1-21.3 pmol/L
Autoimmune profile		
-RA factor	Negative	
-ANA	Negative	
-Anti-deamidated Ab	Negative	
-Anti-transglutaminase Ab	Negative	
HIV Ab	Negative	
Hepatitis B (HbsAg)	Negative	
Anti-HCV	Negative	

**Figure 1:**
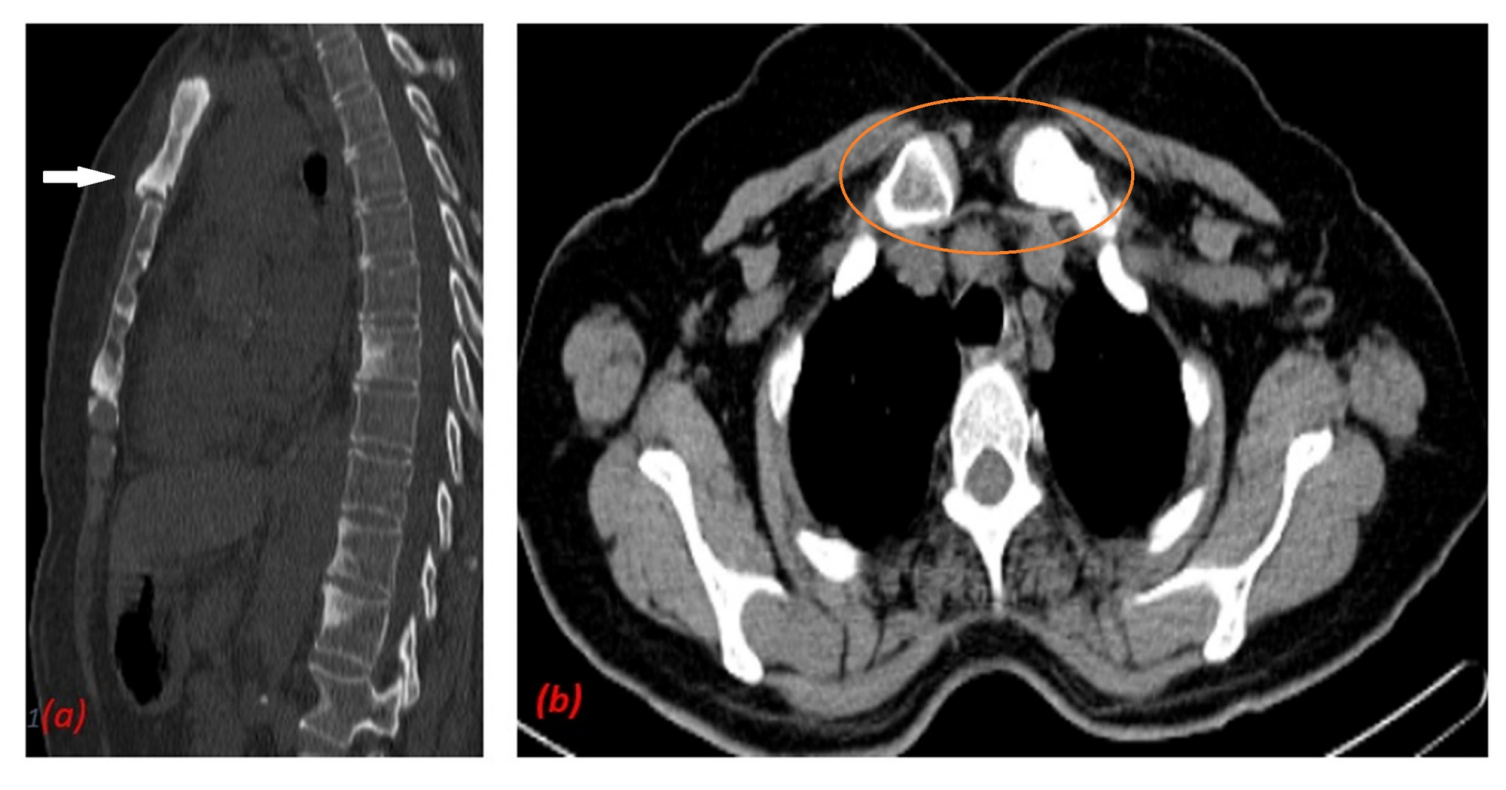
(a) CT scan of the chest shows sclerosis of manubrium (white arrow) and (b) sclerosis of medial part of first ribs (orange circle).

CT scan (Figure 1a) of the chest showed sclerosis and fusion of the manubrium sterni with the medial aspect of first ribs. Additionally, they appeared to be enlarged in size (Figure 1b).

**Figure 2:**
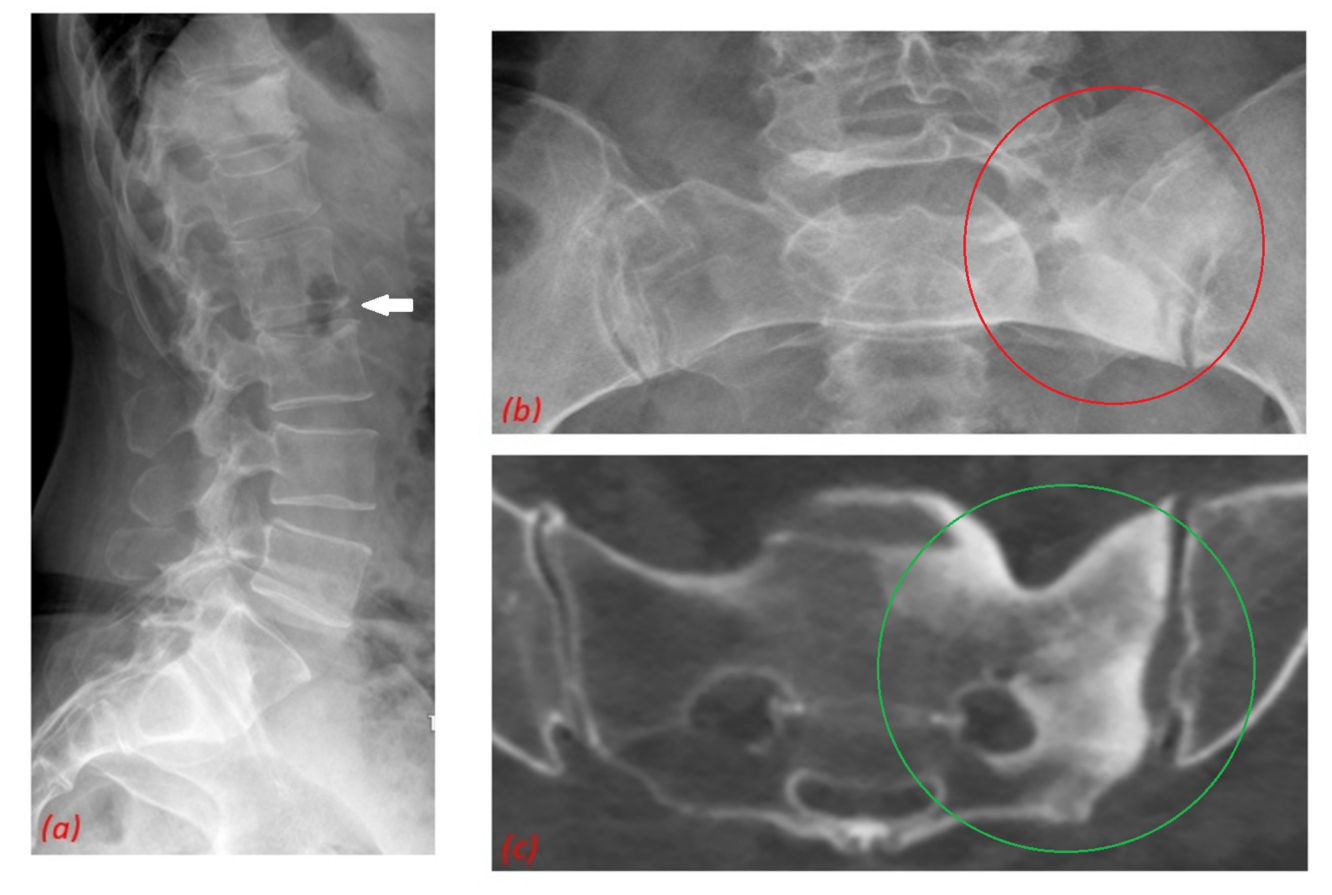
(a) X-ray of the spine shows compression fracture of L3 vertebrae (white arrow). Both X-ray (orange circle) (b) and CT scan (green circle) of the (c) pelvis show multiple areas of sclerosis on the left side of sacrum.

X-ray of the spine (Figure 2a) showed a compression fracture of L3 and vertebral spondylosis of L5 vertebra. Both X-ray (Figure 2b) and CT of the pelvis (Figure 2c) showed multiple areas of sclerosis of the left side of the sacrum. These bony changes suggested the diagnosis of SAPHO syndrome.

**Figure 3:**
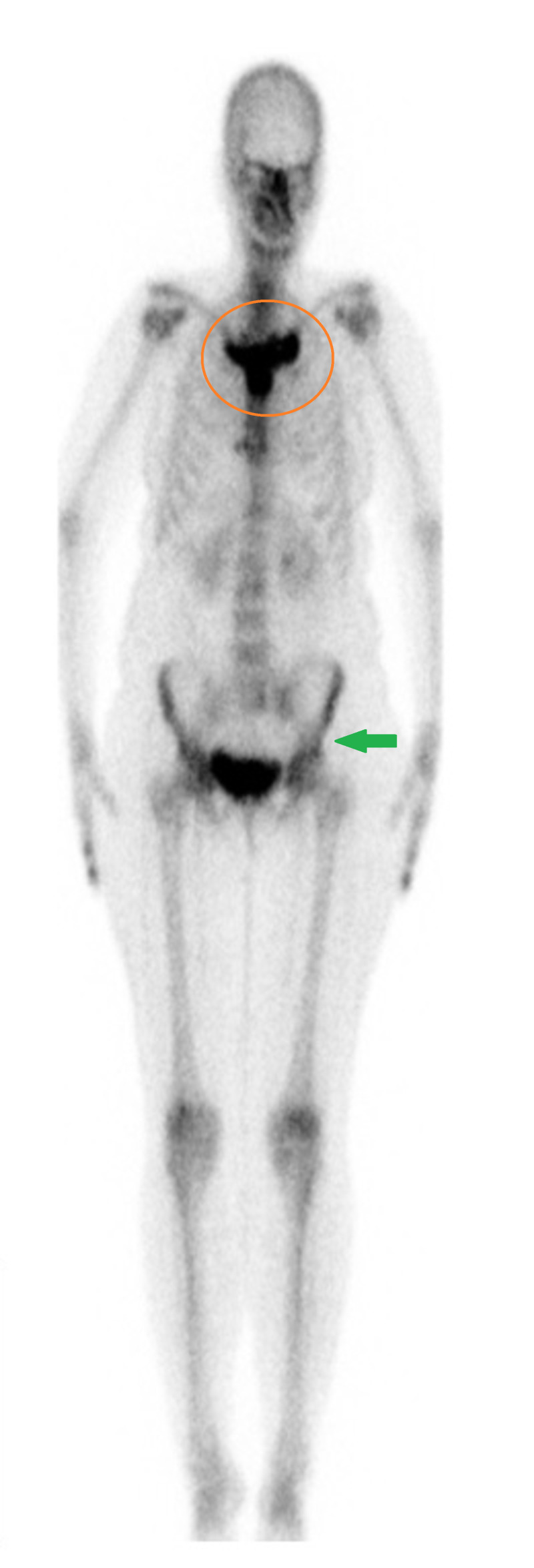
Whole body scan shows classical bullhead appearance (orange circle). Additionally, left sacroiliac joint shows sclerosis (green arrow).

Her whole-body scan (Figure 3) also showed classical “bullhead” appearance and sclerosis of sternoclavicular, manubriosternal and left sacroiliac joints, which matched the areas of sclerosis on CT. These findings were not suggestive of metastatic disease. On the basis, her clinical presentation, baseline blood investigations and supportive radiological evidence, diagnosis of SAPHO syndrome was made. 

## Discussion

Due to its rarity, diagnosis of SAPHO may be challenging. There is wide variation in clinical manifestation and homology of this syndrome in terms of clinical and radiological features with other well-defined disease entities. Furthermore, clinical signs may not be present concurrently and hence may not be correlated that can lead to delayed diagnosis.

Diagnosis

There are several diagnostic criteria’s for SAPHO, of which modified Kahn criteria is shown in Table 2. However, diagnosis of SAPHO remains largely clinical with further augmentation from radiological evidence.

**Table 2:** Table illustrates modified Kahn criteria Modified in 2003 (From Kahn; American College of Rheumatology 67th Annual Scientific Meeting, October 2003) * Exception: growth of Cutibacterium (Propionibacterium) acnes PPP, palmoplantar pustulosis; SAPHO, synovitis, acne, pustulosis, hyperostosis, osteitis

Diagnostic criteria for SAPHO syndrome diagnosis
Inclusion
Bone-joint involvement associated with PPP and psoriasis vulgaris
Bone-joint involvement associated with severe acne
Isolated sterile* hyperostosis/osteitis
Chronic recurrent multifocal osteomyelitis (children)
Bone-joint involvement associated with chronic bowel diseases
Exclusion
Infectious osteitis
Tumoural condition of the bone
Non-inflammatory condensing lesions of the bone

In the majority of patients, multiple joints are affected at presentation. Few joints may be subclinically involved but have radiological evidence suggestive of SAPHO [6]. There can be symmetric involvement, especially in the anterior chest wall, but sarcoileitis is frequently unilateral. The usual clinical course may mimic the pattern of relapsing and remitting rheumatologic flare-up. Typically, there is a slow progression of the osteoarticular manifestations at any site, and a high proportion of patients do not experience long-term debilitating sequelae [3].

Presenting symptoms of affected sites include pain, swelling, tenderness and limited range of movement. Patients tend to be afebrile with normal white blood cell count and C-reactive protein levels, but the ESR is usually raised [7].

Cutaneous manifestations of the disease are highly variable, with an unpredictable course and progression. The most common skin manifestations are those of palmoplantar pustulosis (PPP) and severe acne. Females presenting more commonly with PPP and males more commonly with severe acne [4,5]. Pustular psoriasis is another skin condition with a recognized association with SAPHO.

Radiological imaging

Radiological examination is helpful not only in making a diagnosis but also in the progression and monitoring of the disease. Radiographically, hyperostosis appears as osteosclerosis, with thickening of trabeculae and cortex along with narrowing of the medullary canal [8]. Early lesions tend to be destructive, whereas later lesions tend to be osteoproliferative [9]. X-ray has low sensitivity for the diagnosis of SAPHO [3-5]. Both multidetector computed tomography (MDCT) and bone scintigraphy have vital roles in the diagnosis of SAPHO. MDCT is the primary modality of choice for diagnosis, whereas scintigraphy helps to evaluate the disease activity [10]. On bone scintigraphy, high uptake of the sternoclavicular joints called “bullshead sign” is considered virtually pathognomonic of SAPHO [11]. Whole-body MRI can highlight bone and soft tissue oedema detecting subclinical and radiographically occult sites, and has an advantage of being radiation free. It can be used for follow-up if repeated imaging is required [12]. F-18 fluorodeoxyglucose-positron emission tomography/CT (FDG-PET/CT) can help to differentiate between active and inactive SAPHO lesions, being identified as quiescent and hot scintigraphy areas. The standard uptake value (SUV) of increased uptake areas can also be used to differentiate inflammatory and neoplastic lesion, excluding the possibility of metastatic disease [13].

Differential diagnosis

Bone infections and neoplasia, two differential diagnoses, are important to be excluded prior to consider the diagnoses of SAPHO. However, most often there are clinical and radiologic findings typical of SAPHO, which may help to direct towards the correct diagnosis.

Treatment

In the absence of randomized trials and paucity of high-quality treatment data, there is no single optimal approach to the treatment of SAPHO syndrome. Treatment is based mostly on information gathered from case reports, series or expert opinion. However, the availability of biological agents and the preference of patients should be considered throughout the course of treatment.

Osteoarticular pain of SAPHO is often managed with non-steroidal anti-inflammatory drugs (NSAIDs) or steroids. NSAIDs are usually used as a first-line treatment for analgesia. Intra-articular and systemic glucocorticoids are only transiently effective in most patients, with relapses of both skin and bone involvement at dose reduction or withdrawal [14].

Disease-modifying anti-inflammatory rheumatological drugs (DMARDs) in the form of both biological and non-biological therapies are commonly used as second-line agents. There are controversial results of the use of immunosuppressive drugs such as methotrexate, sulfasalazine, cyclosporine, leflunomide and colchicine on both cutaneous and osteoarticular manifestations [5].

Biological agents (tumour necrosis factor [TNF] inhibitors such as etanercept, adalimumab, infliximab) are typically used after methotrexate failure, and their use is based largely on case reports [15]. Bisphosphonates, especially pamidronate, demonstrated consistent efficacy on bone and osteoarticular manifestations of disease but failed to improve cutaneous lesions [16]. Cutibacterium acnes is speculated to have a role in pathogenesis of the disease; therefore, antibiotics like doxycycline is used as an alternative treatment [17].

## Conclusions

SAPHO syndrome is often a difficult diagnosis, but careful history supported with radiological imaging and clinical suspicion of this syndrome can help the clinician to clinch a diagnosis. Further research is needed to understand the aetiopathogenesis of disease and formulation of definitive treatment by means of randomized trials.
